# Cost-effectiveness of insulin degludec/insulin aspart versus biphasic insulin aspart in Chinese population with type 2 diabetes

**DOI:** 10.3389/fpubh.2022.1016937

**Published:** 2022-10-18

**Authors:** Qiong Luo, Li Zhou, Naitong Zhou, Ming Hu

**Affiliations:** West China School of Pharmacy, Sichuan University, Chengdu, China

**Keywords:** insulin degludec/insulin aspart, Biphasic insulin aspart 30, cost-effectiveness analysis, type 2 diabetes mellitus, China

## Abstract

**Objective:**

To evaluate the long-term cost effectiveness of insulin degludec/insulin aspart (IDegAsp) vs. biphasic insulin aspart 30 (BIAsp 30) for the treatment of people with type 2 diabetes mellitus (T2DM) inadequately managed on basal insulin in China.

**Methods:**

The CORE (the Center for Outcomes Research) Diabetes Model, which has been published and verified, was used to simulate disease progression and calculate the total direct medical costs, life years (LYs) and quality-adjusted life years (QALYs) over 30 years, from the perspective of Chinese healthcare system. The patient demographic information and clinical data needed for the model were gathered from a phase III treat-to-target clinical trial (NCT02762578) and other Chinese cohort studies. Medical costs on treating diabetes were calculated based on clinical trial and local sources. The diabetes management and complications costs were derived from published literature. A discounting rate of 5% was applied to both health and cost outcomes. And one-way and probabilistic sensitivity analyses were carried out to test the reliability of the results.

**Results:**

Compared with BIAsp 30, treatment with IDegAsp was associated with an incremental benefit of 0.001 LYs (12.439 vs. 12.438) and 0.280 QALYs (9.522 vs. 9.242) over a 30-year time horizon, and increased CNY (Chinese Yuan) 3,888 (390,152 vs. 386,264) for total costs. IDegAsp was cost-effective vs. BIAsp 30 therapy with an incremental cost-effectiveness ratio of CNY 13,886 per QALY gained. Results were robust across a range of sensitivity analyses.

**Conclusion:**

Compared with BIAsp 30, IDegAsp was a cost-effective treatment option for people with T2DM with inadequate glycemic management on basal insulin in China.

## Introduction

Diabetes mellitus, a chronic metabolic illness with rising incidence and prevalence globally, has a considerable negative impact on society's financial situation and overall health. The International Diabetes Federation (IDF) estimated that there are 536.6 million people now suffering from diabetes worldwide. In the entire world, China has the largest number of people with diabetes. According to IDF, roughly 140.9 million people aged 20–79 years in China had diabetes in 2021 and annual fatalities from diabetes were estimated to reach over 1.4 million (1). While diabetes and its multiple complications seriously endanger patients' health, they also bring heavy economic burden to patients' families and society. The estimated global direct health expenditure on diabetes in 2021 is USD 966 billion. The United States of America, China and Brazil has the highest estimated expenditure with USD 379.5 billion, USD 165.3 billion and USD 42.9 billion, respectively (1).

Approximately 90% of people with diabetes are type 2 diabetes mellitus (T2DM) (2). Although people with T2DM do not need insulin to maintain life, insulin is still needed to manage hyperglycemia and reduce the risk of complications when the effect of oral hypoglycemic drugs is not good or there is contraindication of oral drug use, and nearly 36% of people with T2DM is treated with insulin (3). Depending on the person with diabetes, basal insulin or premixed insulin (with or without oral antidiabetic drugs) can be used to initiate insulin therapy. To intensify therapy, basal-bolus therapy or premixed insulin in one to three doses is often used. However, all of them have problems such as high number of injections and inconvenience of use.

Biphasic insulin aspart 30 (BIAsp 30) is a widely used premixed insulin, which is a mixture of 30% soluble insulin aspart and 70% protamine zinc insulin aspart. Insulin degludec/insulin aspart (IDegAsp) is a soluble blend of 30% rapid-acting insulin aspart and 70% long-acting insulin degludec, offering both mealtime glycemic management and a long-lasting basal glucose-lowering action. Furthermore, IDegAsp gives people with diabetes ease of use since there is no requirement for resuspension before injection (4).

China has implemented a new centralized medicine procurement policy named the national volume-based procurement (NVBP) since 2018, which try to decrease drug prices through competitive bidding, bulk purchasing, and lower transaction costs. Seven batches of NVBP have been carried out so far, and the sixth batch of NVBP was specifically for insulin procurement and carried out in September 2021. As a result of this insulin procurement, 42 insulin from 11 enterprises were selected, and the average price of selected products was reduced by 48%, with the highest drop of more than 70% (5). BIAsp 30, as a commonly used insulin, had a significant drop in price after NVBP. IDegAsp was included in the National Medical Insurance Medicine Catalog through the negotiation of medical insurance with reduced price in 2020, and the previous cost-effectiveness analysis proved the pharmacoeconomic advantages of IDegAsp (6).

In the past 20 years, diabetes research in China has made significant progress. However, high-quality health economic evaluation is still in high demand (7). It is critical to incorporate clinical and economic evidence when making treatment decisions, so that healthcare providers can maximize resource usage and optimize care for people with T2DM. The efficacy and safety of IDegAsp have been compared with those of BIAsp 30 in three studies involving insulin-naive or insulin-experienced patients with T2DM (8–10). From the results of these studies we know that IDegAsp provides effective overall glycaemic control comparable to BIAsp 30. However, there is not enough analysis of cost-effectiveness among them, particularly from the Chinese perspective. Based on the background of NVBP, the economic burden of insulin use for people with diabetes in China will be further reduced, it is necessary to evaluate the economics of IDegAsp from the perspective of China's health system, so as to provide valuable reference for decision makers in the choice of insulin treatment for T2DM in China.

The aim of this study is to estimate the long term cost-effectiveness of twice-daily IDegAsp vs. twice-daily BIAsp 30 based on the phase III study, from a Chinese healthcare system perspective.

## Materials and methods

### Model overview

We used a proven computer simulation model that called the Center for Outcomes Research (CORE) model to simulate the long-term health outcome and direct medical costs of IDegAsp and BIAsp 30 in the treatment of T2DM. The CORE model is a computer simulation model for both type 1 and type 2 diabetes that has been validated by 66 previously published studies (11). Compared with other long-term models, the CORE model is more suitable for Asian populations because of a new risk equation based on the study of the Hong Kong diabetes registry (HKDR). And it is the most widely used model for insulin economic evaluation (12). The CORE model consists of 17 interdependent Markov submodels and simulates diabetes and its complications using Monte Carlo simulation techniques. The model takes into account baseline cohort characteristics, history of complications, current and future diabetes management, screening strategies, and changes in physiological parameters over time to predict outcomes such as the development of complications, life expectancy, quality-adjusted life years (QALYs), and total cost in the population (13).

### Patient population

A hypothetical simulation cohort was defined based on baseline demographics of Chinese people with T2DM in a phase III, open-label, 2:1 randomized, treat-to-target trial (14), supplemented with data from the literature (15–23) if necessary. This clinical trial was conducted in 40 hospitals from May 2016 to June 2017 in China and included 543 patients, aiming to assess the efficacy and safety of IDegAsp twice-daily vs. BIAsp 30 twice daily in Chinese people with T2DM whose blood glucose levels weren't lower enough by premixed/self-mixed or basal insulin ± metformin. All inputs for Chinese simulation cohort with T2DM is shown in Appendices Table 1.

### Clinical and treatment efficacy inputs

We considered the following treatment effects in our analysis: the changes from baseline in HbA1c, total confirmed hypoglycaemic episodes, nocturnal confirmed hypoglycaemic episodes and severe hypoglycaemic episodes. The treatment effects were derived from the clinical trial (14), Appendices Table 2 lists the treatment effect input variables of two groups.

### Cost inputs

Cost-effectiveness analysis was carried out from the Chinese healthcare system's perspective, which included patients' management costs, treatment costs for diabetes and diabetes-related complications. The costs of treating diabetes mainly includes insulin costs and needle costs. The insulin costs were calculated as its winning bid price times its annual dose. The winning bid price was sourced from national volume-based procurement (NVBP) of insulin and the latest national negotiation, and the insulin dose was obtained from the RCT (14). The annual treatment costs of IDegAsp and BIAsp 30 was 7,832 and 5,700 Chinese Yuan (CNY), respectively. The management costs and diabetes-related complications costs are listed in Appendices Table 3, were mainly based on published literature (21, 24), and all costs were inflated to 2021 CNY using the Chinese Consumer Price Index (25).

### Discounting and time horizon

All expenses and clinical benefits were discounted at a discount rate of 5% per year in compliance with recommendations by Chinese pharmacoeconomic guidelines (26). The 2021 annual gross domestic product (GDP) per capita, was used as the willingness to pay in this study, which was assessed at CNY 80,976 per QALY (25). Given that diabetes is a lifelong chronic disease, we simulated disease progression and health outcomes for patients over 30 years to obtain long-term economic outcomes.

### Utility inputs

Health state utility and event disutility values for type 2 diabetes and its complications were extracted from the literature as shown in Appendices Table 4 (27–29). The utility values were obtained from the literature on utility values in Chinese or Asian populations. For those utility values that cannot be obtained from the literature, the default values of the CORE model (V9.0) were used.

### Sensitivity analysis

One-way sensitivity analysis (OWSA) and probabilistic sensitivity analysis (PSA) were performed to evaluate the effect of changing important input parameters on results. The varying parameters of OWSA included discounting rate (0, 3 or 10%), time horizon (10, 20, 40, and 50 years) and the daily dose of insulin which was adjusted based on a real-world research (22). The PSA performed 1,000 simulations by using a nonparametric bootstrapping approach.

## Results

### Base-case analysis

In the base case analysis (Table 1), the IDegAsp was linked with marginally better discounted life expectancy of 0.001 years per patient compared with the BIAsp 30 (12.439 vs. 12.438 years). Similar benefits of the IDegAsp were demonstrated on life quality. It was associated with an incremental benefit of 0.280 QALYs per patient compared with the BIAsp 30 (9.522 vs. 9.242 QALYs).

**Table 1:** Base-case analysis.

**Parameter**	**IDegAsp**	**BIAsp 30**	**Relative difference**
Life expectancy (years)	12.439	12.438	0.001
Quality-adjusted life expectancy (QALYs)	9.522	9.242	0.280
Direct medical costs (CNY)	390,152	386,264	3,888
Treatment	99,676	72,537	27,139
Patient management^I^	26,966	26,982	−16
Complication costs			
Cardiovascular disease	153,538	153,215	323
Renal disease	4,428	4,409	19
Ulcer, amputation and neuropathy	76,711	77,085	−374
Eye disease	2,402	2,434	−32
Hypoglycemia	26,431	49,602	−23,171
ICER based on quality-adjusted life expectancy		13,886	

I The management costs were reported as annual costs including hospitalization, daily medications (non-hypoglycemic agents) and examinations for DM-related chronic complications (21).

The IDegAsp cohort predicted lower cumulative incidence of all diabetes-related complications compared with that of the BIAsp 30 cohort (Table 2). Notable reductions were projected for non-severe and severe hypoglycemia. Besides, there were slight reductions in the incidence of all eye complications, renal complications, foot complications and cardiovascular complications.

**Table 2:** Cumulative incidence of diabetes-related complications over 30 years.

**Parameter**	**IDegAsp**	**BIAsp 30**	**Relative difference**
Eye complications (%)			
Background retinopathy	33.39	33.63	−0.24
Proliferative retinopathy	11.55	11.63	−0.08
Macular oedema	28.48	28.69	−0.21
Severe vision loss	16.52	16.71	−0.19
Cataract	13.61	13.70	−0.09
Renal complications (%)			
Microalbuminuria	42.21	42.31	−0.10
Gross proteinuria	21.75	21.85	−0.10
End-stage renal disease	9.94	9.99	−0.05
Foot complications (%)			
Foot ulcer (first)	51.52	51.63	−0.11
Foot ulcer (recurrence)	123.56	124.04	−0.48
Amputation (first)	25.98	26.07	−0.09
Amputation (recurrence)	10.00	10.06	−0.06
Neuropathic complications (%)			
Neuropathy	78.92	79.03	−0.11
Cardiovascular complications (%)			
Congestive heart failure	11.82	11.92	−0.10
Peripheral vascular disease	23.29	23.34	−0.05
Angina	14.92	15.02	−0.10
Stroke	11.22	11.29	−0.07
Myocardial infarction	22.12	22.16	0.04
Hypoglycaemic episodes (events/patient)			
Non-severe hypoglycemia	53.47	90.47	−37.00
Severe hypoglycemia	0.0	5.31	−5.31

Calculations of direct medical costs for patients over 30 years showed that the average cost of IDegAsp group was CNY 3,888 higher than BIAsp 30 group. IDegAsp was associated with increased treatment costs, but it also resulted in savings in complication costs.

For people with T2DM, 30-year period's estimation showed that, treatment with IDegAsp was the more economical choice compared with BIAsp 30. The estimated Incremental Cost-Effective Ratio (ICER) in the base-case analysis was CNY 13,886 per QALY gained, under the threshold of the gross domestic product (GDP) per capita in China.

### Sensitivity analysis

The results of a series of OWSA demonstrated that varying discounting rate, time horizons and the daily dose of insulin had no significant effect on final results. In the scenarios that the daily dose of insulin was adjusted based on the real-world research, IDegAsp was a dominant choice because of higher QALYs and lower costs compared with BIAsp 30. In other scenarios of the sensitivity analysis, IDegAsp was associated with increased QALYs and increased costs compared with BIAsp 30, the ICER was always below the threshold. Changes in key parameters did not change the economic results that the treatment with IDegAsp was the cost-effective choice (Table 3).

**Table 3:** One-way sensitivity analyse.

**Sensitivity analysis**	**Quality-adjusted life expectancy (QALYs)**			**Total costs (CNY)**			**ICER**
	**IDegAsp**	**BIAsp 30**	**Difference**	**IDegAsp**	**BIAsp 30**	**Difference**	
0% discount rates	16.349	15.868	0.481	748,926	741,776	7,150	14,865
3% discount rates	11.592	11.251	0.341	495,500	490,651	4,849	14,220
8% discount rates	7.381	7.164	0.217	285,908	282,979	2,929	13,498
10-year time horizon	5.843	5.673	0.170	192,449	190,544	1,905	11,206
20-year time horizon	8.566	8.303	0.263	325,161	322,240	2,921	11,106
40-year time horizon	9.773	9.476	0.297	410,597	406,522	4,075	13,720
50-year time horizon	9.817	9.522	0.295	415,878	412,199	3,679	12,471
Daily dose of insulin	9.522	9.242	0.280	348,253	359,848	−11,595	−41,411

**Figure 1:**
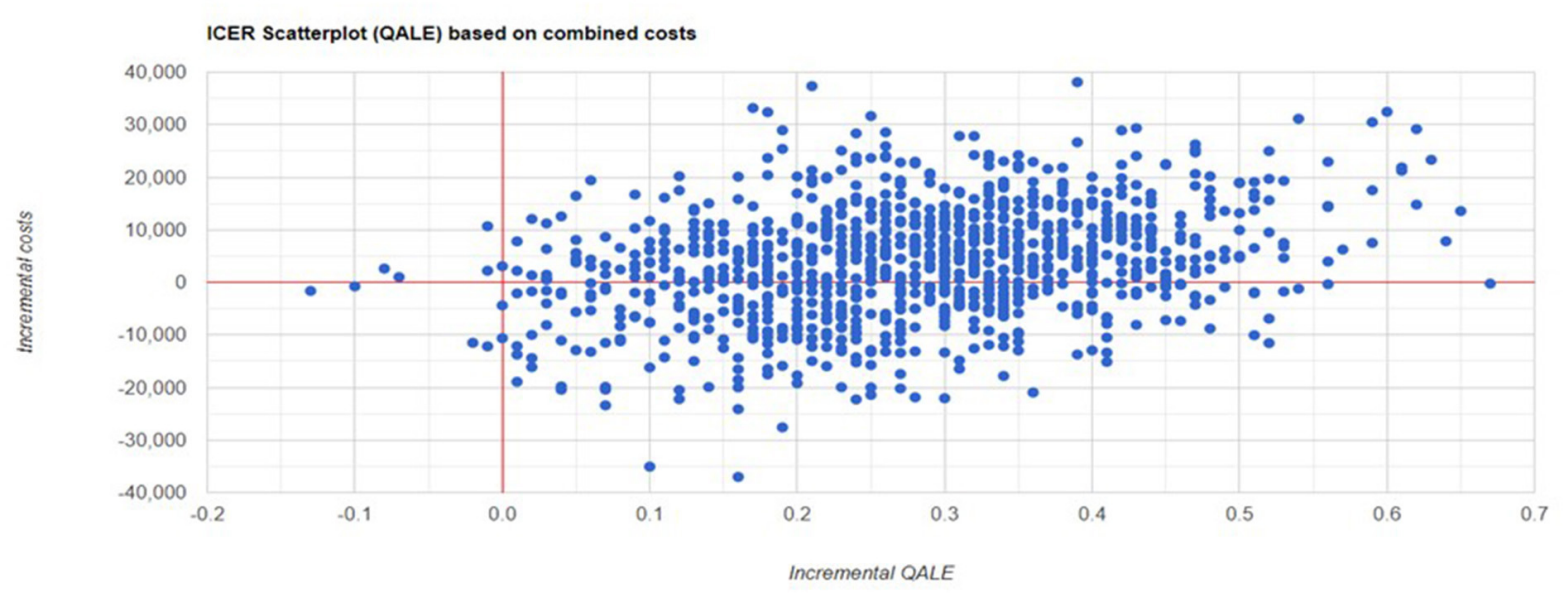
Scatter plots for probability sensitivity analyses.

**Figure 2:**
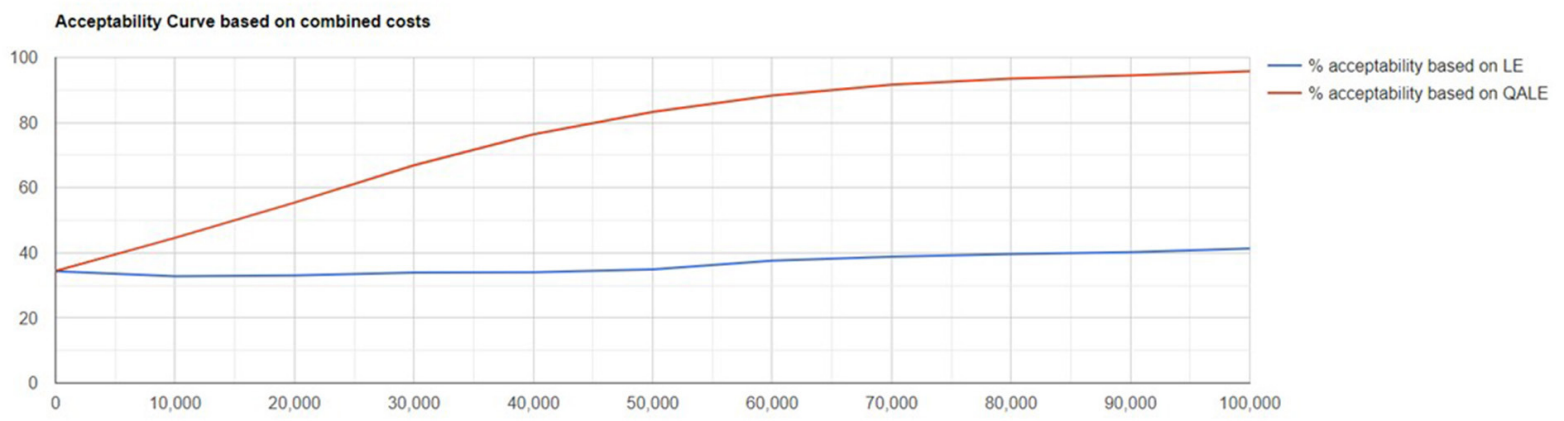
Cost-effectiveness acceptability curve.

Probabilistic sensitivity analysis showed the results were robust to variable changes. Scatter plot of change in costs vs. change in QALYs and cost-effectiveness acceptability curve are showed in Figures 1, 2.

## Discussion

Diabetes is a chronic metabolic disease with a long duration, which not only seriously harms the health of patients, but also places a significant financial burden on them and their families. When choosing treatment options for people with diabetes, it is necessary to consider not only the short-term effects of treatment options, but also the long-term health outcomes and health costs. Despite the fact that there were numerous oral drugs available to lower blood glucose levels, the majority of people with T2DM in China did not have their blood glucose levels sufficiently under control. Approximately 36% of individuals eventually require insulin to keep their HbA1c levels optimal (30).

In this study, the CORE model was applied to simulate long-term disease progression and health outcomes in patients treated with IDegAsp and BIAsp 30 in the Chinese setting. We have identified that, compared with BIAsp 30, the IDegAsp regimen resulted in more life years and QALYs, while also generating higher direct medical costs, based on the clinical trial and our modeling analysis. In order to verify the robustness of the results, the time horizons were adjusted according to the difference between the average life span of the Chinese population and the clinical trial, and different discounting rates were used according to the guidelines, and the daily dose of insulin was adjusted on the basis of real-world evidence considering the difference in insulin dose between the real world and clinical trials to conduct sensitivity analysis. The result's robustness was further supported by OWSA and PSA, which demonstrated that IDegAsp was more economical than BIAsp 30.

According to the results, the incremental benefit of LYs is small. The higher initial age of the simulation cohort may have contributed to the small difference in LYs. We thus ran a sensitivity analysis of the time horizon, and the results were robust and did not reverse. In addition, a slight distinction in QALYs is likely to be due to the clinical trials on which the model simulations are based. We used the results of a treat-to-target trial for long-term simulation. This trial demonstrated non-inferiority of IDegAsp vs. BIAsp 30 for the change from baseline to week 26 in HbA1c. And the change in HbA1c is one of the input parameters of the CORE model, based on which the CORE model predicts outcomes such as QALYs. Finally, diabetes is a chronic disease, and the incremental QALYs brought by hypoglycemic agents are generally not large. A recent long-term cost-effectiveness study suggested that Dapagliflozin plus standard treatment was anticipated to produce an additional 0.25 QALYs in comparison to standard treatment (31). Another cost-effectiveness analysis of iGlarLixi (insulin glargine 100 U/mL plus lixisenatide) vs. iDegLira (insulin degludec plus liraglutide) demonstrated an incremental QALY of 0.015 (32).

A cost-effectiveness comparison of IDegAsp with BIAsp 30 has been performed. Earlier, Marc Evans conducted a cost-effectiveness analysis of IDegAsp compared with BIAsp 30 by using a simple and transparent short-term model in Denmark (33). Similar to our study, this short-term cost-effectiveness analysis demonstrates that IDegAsp is a cost-effective option compared with BIAsp 30 for T2DM patients. Obviously, the costs included in the short-term and long-term simulations are different, with the long-term simulations taking more into account the costs of treating complications. Because of the different emphasis of short-term and long-term simulation, it is more comprehensive to combine the two resultsfor evaluation.

This analysis has several strengths. Many of the latest data, including price and utility values, were used to ensure that this economic evaluation was based on the latest evidence, which satisfies the requirement that health economic evaluation be continuously updated and support medical decision-making. A number of sensitivity analyses were conducted and found that the results were robust to changes in crucial assumptions. But we should be aware of several limitations in our analysis. First, there were many disease states involved in the CORE model, the utility values of different disease states and transition probabilities used in the CORE model were obtained from epidemiological research and clinical trials conducted primarily in Western populations. There may be some differences in disease utility and disease progression from the Chinese population. This was a flaw in the modeling analysis. Second, in the absence of long-term studies in China, the CORE model simulated long-term clinical outcomes based on a 26-week randomized trial. Given the chronic pattern of T2DM, the lengths of the cited clinical trials' treatments may be too short to simulate long-term efficacy perfectly. Bisides, only direct medical costs were included in this study. Direct non-medical costs such as transportation expenses and indirect costs associated with lost productivity were not included. Considering that IDegAsp is more economical than BIAsp 30 in these two types of costs due to its lower incidence of complications, the overall benefit of IDegAsp therapy may be underestimated. Finally, the utility value data in this study was primarily derived from 2 studies because there were few complete and accessible studies on the utility value of people with diabetes in the Chinese population. It is best to improve after there is a better utility value study in the future.

## Conclusion

In conclusion, this study suggests that IDegAsp was a cost-effective treatment option for people with T2DM from the Chinese healthcare system's perspective. Our findings may help clinicians make better decisions about diabetes treatment.

## Data availability statement

The original contributions presented in the study are included in the article/Supplementary material, further inquiries can be directed to the corresponding authors.

## Author contributions

LZ and QL were involved in the study design, data collecting, analysis, and interpretation, as well as the authoring and revision of this manuscript. MH and NZ reviewed the final model design, data sources, and results. All authors contributed to the article and approved the submitted version.

## Funding

This study was supported by Novo Nordisk (China). The funding agencies had no role in the study design, data collection or analysis, decision to publish, or preparation of the manuscript.

## Conflict of interest

The authors declare that the research was conducted in the absence of any commercial or financial relationships that could be construed as a potential conflict of interest.

## Publisher's note

All claims expressed in this article are solely those of the authors and do not necessarily represent those of their affiliated organizations, or those of the publisher, the editors and the reviewers. Any product that may be evaluated in this article, or claim that may be made by its manufacturer, is not guaranteed or endorsed by the publisher.
