# Supplementary material for: Cost-effectiveness of insulin degludec/insulin aspart versus biphasic insulin aspart in Chinese population with type 2 diabetes
Source: Front Public Health. 2022 Oct 18;10:1016937. doi: 10.3389/fpubh.2022.1016937 (PMC9623119; doi:10.3389/fpubh.2022.1016937)
Supplement: Supplementary file 1 [file Table_1.DOCX]

**Appendices**

**Appendices Table 1 Characteristics and complications of simulated cohort**

**Appendices Table 2 Treatment effects applied in the analysis**

**Appendices Table 3 Annual management and diabetes-related complication costs**

**Appendices Table 4 Utility values for each health state applied in the analysis**

**Appendices Table 1 Characteristics and complications of simulated cohort**

| Cohort | Mean ± SD | Source |
| --- | --- | --- |
| Demographics |  |  |
| Age (years) | 59.4±9.2 | (1) |
| Duration of diabetes (years) | 12.8±6.4 | (1) |
| Men, n (%) | 54.7 | (1) |
| Baseline risk factors |  |  |
| HbA1c (%) | 8.3±0.8 | (1) |
| Systolic pressure (mmHg) | 131.1±15.5 | (2) |
| Diastolic blood pressure (mmHg) | 79.8±9.2 | (2) |
| Cholesterol (g/L) | 189.87±41.76 | (3) |
| HDL cholesterol (g/L) | 51.04±13.53 | (3) |
| LDL cholesterol (g/L) | 105.57± 34.03 | (3) |
| Triglycerides (g/L) | 177.15±149.69 | (3) |
| BMI (kg/m^2^) | 25.5±3.3 | (2) |
| eGFR (ml/min/1.73m^2^) | 104.9 | (4) |
| Blood haemoglobin (gr/dl) | 13.8 | (4) |
| White blood cell count (10^9^/counts/L) | 7 | (4) |
| Heart rate (bpm) | 72±12 | (5) |
| Waist–hip rate | 0.93 | (5) |
| Spot urinary albumin creatinine ratio (mg/mmol) | 2 | (4) |
| Serum creatinine (mg/dl) | 1.1 | (5) |
| Serum albumin (g/dl) | 3.9 | (5) |
| Proportion smokers (%) | 27.50 | (6) |
| Cigarettes (n/days) | 15.2 | (7) |
| Alcohol consumption (oz./week) | 4.36 | (5) |
| Baseline cardiovascular disease complications (%) |  |  |
| Proportion with myocardial infarction | 2.15 | (8) |
| Proportion with angina | 1.72 | (8) |
| Peripheral vascular disease | 17.00 | (9) |
| Proportion with stroke | 5.70 | (9) |
| Proportion with congestive heart failure | 0.43 | (8) |
| Proportion with atrial fibrillation | 1.29 | (8) |
| Proportion with left ventricular hypertrophy | 0.00 | (8) |
| Baseline renal complications (%) |  |  |
| Proportion with microalbuminuria | 4.00 | (8) |
| Proportion with gross proteinuria | 4.00 | (8) |
| Proportion with end-stage renal disease | 0.00 | (8) |
| Baseline eye complications (%) |  |  |
| Proportion with background retinopathy | 24.50 | (9) |
| Proportion with proliferative retinopathy | 0.00 | (8) |
| Proportion with severe vision loss | 0.00 | (8) |
| Proportion with macular edema | 0.00 | (8) |
| Proportion with cataract | 4.30 | (8) |
| Baseline foot ulcer complications (%) |  | (8) |
| Uninfected ulcer | 0.43 | (8) |
| Infected ulcer | 0.43 | (8) |
| Healed ulcer | 0.00 | (8) |
| History of amputation | 0.43 | (8) |
| Baseline neuropathic complications (%) |  |  |
| Proportion with neuropathy | 22.10 | (10) |
| Baseline depression (%) |  |  |
| Proportion with depression | 0.00 | (8) |

Abbreviations: HDL, high-density lipoprotein; LDL, low-density lipoprotein; eGFR, estimated glomerular filtration rate

**Appendices Table 2 Treatment effects applied in the analysis**

| Parameter | IDegAsp | BIAsp 30 |
| --- | --- | --- |
| Change from baseline HbA1c (%), mean ± SD | −1.48±0.05 | −1.40±0.06 |
| Overall hypoglycaemia (events/100 PYE) | 237.16 | 412.16 |
| Nocturnal hypoglycaemia (events/100 PYE) | 34.86 | 61.02 |
| Severe hypoglycaemia (events/100 PYE) | 0 | 10.36 |

Abbreviations: PYE, patient–years of exposure

**Appendices Table 3 Annual management and diabetes-related complication costs**

| Parameter | Costs (CNY) | Source |
| --- | --- | --- |
| Management costs |  |  |
| Statins | 2,841 | (8) |
| Aspirin | 226 | (8) |
| Angiotensin-converting enzyme inhibitors | 1,653 | (8) |
| Microalbuminuria screening | 166 | (8) |
| Gross proteinuria screening | 71 | (8) |
| Eye screening | 409 | (8) |
| Foot screening | 1,468 | (8) |
| Anti-depression treatment | 2,380 | (8) |
| Screening for depression | 92 | (8) |
| Direct costs: CVD complications |  |  |
| Cost of myocardial infarction, first year | 22,813 | (11) |
| Cost of myocardial infarction, subsequent years | 24,286 | (11) |
| Cost of angina, first year | 23,238 | (11) |
| Cost of angina, subsequent years | 25,100 | (11) |
| Cost of coronary heart failure, first year | 28,506 | (11) |
| Cost of coronary heart failure, subsequent years | 26,530 | (11) |
| Cost of stroke, first year | 24,415 | (11) |
| Cost of stroke, subsequent years | 19,226 | (11) |
| Cost of stroke, death within 30 days of event | 30,154 | (8) |
| Cost of peripheral vascular disease, first year | 24,517 | (11) |
| Cost of peripheral vascular disease, subsequent years | 32,279 | (11) |
| Direct costs: renal complications |  |  |
| Cost of haemodialysis, first year | 20,186 | (11) |
| Cost of haemodialysis, subsequent years  Cost of peritoneal dialysis, first year | 23,612  65,106 | (11) |
|  |  | (8) |
| Cost of peritoneal dialysis, subsequent years | 53,104 | (8) |
| Cost of renal transplant, first year | 281,826 | (8) |
| Cost of renal transplant, subsequent years | 74,555 | (8) |
| Direct costs: acute events |  |  |
| Cost of severe hypoglycaemic event | 13,652 | (11) |
| Cost of non-severe hypoglycaemic event | 871 | (11) |
| Cost of ketoacidosis event | 12,463 | (8) |
| Cost of lactic acidosis event | 12,617 | (8) |
| Cost of edema, first year | 16,281 | (8) |
| Cost of edema, subsequent year | 2,142 | (8) |
| Direct costs: eye disease |  |  |
| Cost of laser treatment | 2,476 | (8) |
| Cost of cataract operation, first year | 10,962 | (8) |
| Cost of cataract operation, subsequent year | 280 | (8) |
| Cost of blindness, first year | 2,304 | (8) |
| Cost of blindness, subsequent year | 761 | (8) |
| Direct costs: Neuropathy, amputation and ulcer |  |  |
| Cost of neuropathy, first year | 18,582 | (8) |
| Cost of neuropathy, subsequent year | 7,129 | (8) |
| Cost of amputation event | 17,293 | (8) |
| Cost of prosthesis following amputation event | 15,530 | (8) |
| Cost of treating uninfected ulcer | 30,438 | (8) |
| Cost of treating infected ulcer | 25,094 | (8) |
| Cost of standard uninfected ulcer | 20,882 | (8) |

**Appendices Table 4 Utility values for each health state applied in the analysis**

| Health state/events | Value | Source |
| --- | --- | --- |
| T2DM without complication | 0.881 | (12) |
| Myocardial infarction | 0.874 | (12) |
| Angina | 0.864 | (12) |
| Congestive heart failure | 0.831 | (12) |
| Stroke | 0.717 | (13) |
| Peripheral vascular disease | 0.864 | (12) |
| Proteinuria | 0.833 | (13) |
| Hemodialysis | 0.717 | (13) |
| Peritoneal dialysis | 0.677 | (13) |
| Renal transplant | 0.858 | (13) |
| Background retinopathy | 0.858 | (12) |
| Proliferative retinopathy | 0.811 | (13) |
| Macular edema | 0.841 | (13) |
| Severe vision loss | 0.780 | (12) |
| Cataract | 0.865 | (12) |
| Neuropathy | 0.829 | (12) |
| Healed ulcer | 0.881 | (13) |
| Infected ulcer | 0.711 | (13) |
| Amputation | 0.704 | (12) |
| Depression | 0.881 | (13) |
| Non-severe hypoglycaemia | -0.014 | (13) |
| Severe hypoglycaemia | −0.0183 | (14) |
| Very severe hypoglycaemia | -0.047 | (13) |

**References**

1. Yang W, Ma J, Hong T, Liu M, Miao H, Peng Y, et al. Efficacy and Safety of Insulin Degludec/Insulin Aspart Versus Biphasic Insulin Aspart 30 in Chinese Adults with Type 2 Diabetes: A Phase Iii, Open‐Label, 2:1 Randomized, Treat‐to‐Target Trial. *Diabetes Obesity & Metabolism* (2019) 21(7):1652-60. doi: 10.1111/dom.13703.

2. Bo Z, Jing Z, Yang W. Glycemic Control and Safety in Chinese Patients with Type 2 Diabetes Mellitus Who Switched from Premixed Insulin to Insulin Glargine Plus Oral Antidiabetics: A Large, Prospective, Observational Study. *Diabetes/Metabolism Research and Reviews* (2017) 33(3):e2683. doi: 10.1002/dmrr.2863.

3. Jia W, Xiao X, Ji Q, Ahn KJ, Chuang LM, Bao Y, et al. Comparison of Thrice-Daily Premixed Insulin (Insulin Lispro Premix) with Basal-Bolus (Insulin Glargine Once-Daily Plus Thrice-Daily Prandial Insulin Lispro) Therapy in East Asian Patients with Type 2 Diabetes Insufficiently Controlled with Twice-Daily Premixed Insulin: An Open-Label, Randomised, Controlled Trial. *Lancet Diabetes Endocrinol* (2015) 3(4):254-62. doi: 10.1016/S2213-8587(15)00041-8.

4. Yang X, Ma RC, So WY, Kong AP, Ko GT, Ho CS, et al. Development and Validation of a Risk Score for Hospitalization for Heart Failure in Patients with Type 2 Diabetes Mellitus. *Cardiovascular Diabetology* (2008) 7(1):9. doi: 10.1186/1475-2840-7-9.

5. Su W, Li C, Zhang L, Lin Z, Tan J, Xuan J. Meta-Analysis and Cost-Effectiveness Analysis of Insulin Glargine 100 U/Ml Versus Insulin Degludec for the Treatment of Type 2 Diabetes in China. *Diabetes Therapy* (2019) 10(5):1969-84. doi: 10.1007/s13300-019-00683-2.

6. Wang L, Gao P, Zhang M. Prevalence and Ethnic Pattern of Diabetes and Prediabetes in China in 2013. *JAMA* (2017) 317(24):2515-23. doi: 10.1001/jama.2017.7596.

7. Yang Y, Nan Y, Tu M, Wang J, Wang L, Jiang Y. Major Finding of 2015 China Adults Tobacco Survery. *Chinese Journal of Health Management* (2016) 10(2):85-7. doi: 10.3760/cma.j.issn.1674-0815.2016.02.002.

8. Wu J, He X, Liu Y. Cost-Effectiveness Analysis of Insulin Aspart 30 Versus Insulin Glargine in Patients with Type 2 Diabetes in China. *Chinese Pharmaceutical Journal* (2016) 51(3):242-7. doi: 10.11669/cpj.2016.03.021.

9. Yang W, Gao Y, Liu G, Chen L, Fu Z, Zou D, et al. Biphasic Insulin Aspart 30 as Insulin Initiation or Replacement Therapy: The China Cohort of the Improve Study. *Current Medical Research & Opinion* (2010) 26(1):101-7. doi: 10.1185/03007990903364640.

10. Yang W, Ersoy C, Wang G, Ye S, Liu J, Miao H, et al. Efficacy and Safety of Three-Times-Daily Versus Twice Daily Biphasic Insulin Aspart 30 in Patients with Type 2 Diabetes Mellitus Inadequately Controlled with Basal Insulin Combined with Oral Antidiabetic Drugs. *Diabetes Research and Clinical Practice* (2019) 150:158-66. doi: 10.1016/j.diabres.2019.02.023.

11. Duan X, Li C, Li Y, Liu Q. Epidemiological Characteristics, Medical Costs and Healthcare Resource Utilization of Diabetes-Related Complications among Chinese Patients with Type 2 Diabetes Mellitus. *Expert Review of Pharmacoeconomics & Outcomes Research* (2020) 20(5):513-21. doi: 10.1016/j.jval.2018.07.303.

12. Mok CH, Kwok HHY, Ng CS, Leung GM, Quan J. Health State Utility Values for Type 2 Diabetes and Related Complications in East and Southeast Asia: A Systematic Review and Meta-Analysis. *Value in Health* (2021) 24(7):1059-67. doi: <http://dx.doi.org/10.1016/j.jval.2020.12.019>.

13. Beaudet A, Clegg J, Thuresson PO, Lloyd A, Mcewan P. Review of Utility Values for Economic Modeling in Type 2 Diabetes. *Value in Health* (2014) 17(4):462-70. doi: 10.1016/j.jval.2014.03.003.

14. Marrett E, Radican L, Davies MJ, Zhang Q. Assessment of Severity and Frequency of Self-Reported Hypoglycemia on Quality of Life in Patients with Type 2 Diabetes Treated with Oral Antihyperglycemic Agents: A Survey Study. *BMC Research Notes* (2011) 4(1):251-. doi: 10.1186/1756-0500-4-251.
